# High-Yield Precursor-Derived Si-O Ceramics: Processing and Performance

**DOI:** 10.3390/ma18153666

**Published:** 2025-08-04

**Authors:** Xia Zhang, Bo Xiao, Yongzhao Hou, Guangwu Wen

**Affiliations:** 1 School of Chemistry and Chemical Engineering, Shandong University of Technology, Zibo 255000, China; 18409350572@stumail.sdut.edu.cn (X.Z.); xiaobosdut587@sina.com (B.X.); 2 School of Materials Science and Engineering, Shandong University of Technology, Zibo 255000, China; houyz1990@sdut.edu.cn

**Keywords:** pyrolysis, ceramic precursor, anionic ring opening, ceramic yield, wave-transparent

## Abstract

The precursor-derived ceramic route is recognized as an advanced and efficient technique for fabricating ceramic matrix composites, particularly suitable for the development and microstructural tailoring of continuous fiber-reinforced ceramic matrix composites. In this work, octamethylcyclotetrasiloxane and tetravinylcyclotetrasiloxane were employed as monomers to synthesize a branched siloxane via ring-opening polymerization. A subsequent hydrosilylation reaction led to the formation of polyvinylsiloxane with a three-dimensional crosslinked structure. The precursor exhibited excellent fluidity, adjustable viscosity, and superior thermosetting characteristics, enabling efficient impregnation and densification of reinforcements through the polymer infiltration and pyrolysis process. Upon pyrolysis, the polyvinylsiloxane gradually converted from an organic polymer to an amorphous inorganic ceramic phase, yielding silicon oxycarbide ceramics with a high ceramic yield of 81.3%. Elemental analysis indicated that the resulting ceramic mainly comprised silicon and oxygen, with a low carbon content. Furthermore, the material demonstrated a stable dielectric constant (~2.5) and low dielectric loss (<0.01), which are beneficial for enhanced thermal stability and dielectric performance. These findings offer a promising precursor system and process reference for the low-cost production of high-performance, multifunctional ceramic matrix composites with strong potential for engineering applications.

## 1. Introduction

Polymer-derived ceramics (PDCs) represent a versatile and advanced route for the fabrication of inorganic ceramics through the pyrolytic conversion of preceramic polymers [1,2]. This transformation typically involves thermal curing, crosslinking, and subsequent high-temperature pyrolysis, during which the organic functionalities decompose and volatile species are eliminated, leading to the formation of an amorphous or nanostructured ceramic network [3]. The PDC approach offers several distinct advantages, including molecular-level tunability of precursor chemistry, compatibility with diverse shaping techniques, and relatively low ceramic processing temperatures [4]. Owing to these merits, PDCs have been extensively explored for applications in high-temperature structural components, electrical insulation, chemical sensors, and oxidation-resistant coatings. Among various PDC systems, silicon oxycarbide (SiOC) ceramics have garnered considerable attention due to their superior thermal stability, creep resistance, and excellent chemical durability [5]. However, conventional siloxane-based precursors often exhibit significant mass loss during pyrolysis, attributed to the evolution of organic volatiles, which limits the ceramic yield typically to 60–70% [6]. This inherent limitation poses challenges for large-scale manufacturing and restricts broader industrial implementation. Consequently, the development of novel preceramic systems with enhanced ceramic yield while maintaining desirable structural integrity and functional performance remains a central objective in current PDC research [7].

In recent years, polysiloxanes have garnered significant attention as promising precursors for the fabrication of silicon oxycarbide (SiOC) ceramics due to their Si-O-rich backbone and favorable pyrolytic conversion characteristics [8]. Upon pyrolysis, these precursors yield amorphous SiOC ceramics that exhibit excellent thermal and dielectric stability while maintaining structural integrity at elevated temperatures ranging from 1000 °C to 1500 °C [9,10]. A representative high-performance application of such materials is exemplified by the X-37B spaceplane developed by the United States, which incorporates advanced thermal protection systems and structural ceramic components [11]. Notably, the nose cone and windward surfaces of the vehicle are shielded by a novel toughened uni-piece fibrous reinforced oxidation-resistant composite [12]. This lightweight, integrated thermal protection system comprises a high-temperature-resistant thermal cap and an insulating substrate. The thermal cap is composed of refractory oxidation-resistant carbon insulation, whereas the insulating layer is fabricated via polymer infiltration and pyrolysis (PIP) of a SiOC precursor into a carbon fiber preform, followed by application of a thermal barrier coating [13]. Moreover, the microstructure of polysiloxane-derived SiOC ceramics can be effectively tailored by modulating the free carbon content and the architecture of the inorganic network [14]. This tunability enables precise control over dielectric parameters, including the dielectric constant and loss tangent, thereby offering significant promise for applications in electromagnetic wave transparency and stealth technologies [15]. In particular, polysiloxane-based ceramics are highly suitable for the fabrication of fiber-reinforced ceramic matrix composites, which synergistically combine the thermal and mechanical robustness of ceramics with the enhanced toughness imparted by fiber reinforcement [16,17]. Such composites are particularly attractive for use in hypersonic vehicles, thermal protection systems, and radar-transparent structural components [18,19]. During the polymer infiltration and pyrolysis (PIP) process, polysiloxane precursors exhibit excellent infiltration behavior and coating uniformity [20]. Following pyrolysis, the resulting SiOC ceramics demonstrate favorable interfacial compatibility with ceramic fibers such as quartz and SiC, enabling the formation of strong interfacial bonding and contributing to the improved mechanical and functional performance of the composite system [21,22].

Therefore, we propose a novel strategy for the development of high-yield, wave-transparent ceramic composites by synthesizing a liquid polysiloxane precursor with precisely tailored molecular architecture. The approach is based on a structure-directed molecular design that combines ring-opening polymerization and hydrosilylation in a single-step process, resulting in a crosslinkable, thermosetting siloxane network. Unlike conventional sol–gel or oligomer-based systems, our precursor exhibits enhanced infiltration behavior and significantly reduced processing complexity. The unique combination of tunable rheological properties, high ceramic conversion efficiency, and compatibility with fiber-reinforced composite fabrication distinguishes this work from previous studies. This methodology offers a promising route toward the scalable production of low-dielectric, thermally stable ceramic matrix composites for use in high-frequency electromagnetic applications such as radomes, microwave windows, and stealth structures.

## 2. Experimental

### 2.1. Raw Materials

Tetramethylcyclotetrasiloxane (AR, ≥98%, D_4_^H^) and tetravinylcyclotetrasiloxane (AR, ≥98%, D_4_^Vi^) were purchased from Sinopharm, China. Tetra-methylammonium hydroxide (AR, 25% aqueous solution, TMAH) was supplied by Tianjin Guangfu Fine Chemical Research Institute Co., Ltd., Tianjin, China.

### 2.2. Preparation of PVSO

A 1 L three-necked round-bottom flask equipped with a mechanical stirrer, a thermometer, and a dropping funnel was placed in an ice-water bath to maintain the reaction temperature at approximately 0 °C. Equimolar amounts of D_4_^Vi^ and D_4_^H^ were introduced into the flask under ambient atmosphere, ensuring a 1:1 molar ratio of Si-H to vinyl functional groups. This ratio was carefully selected to promote an efficient hydrosilylation reaction and to obtain a uniform network structure in the final product.

The mixture was stirred continuously until a homogeneous and transparent solution was formed, indicating adequate mixing and dissolution of the monomers. Tetramethylammonium hydroxide (TMAH), used as a base catalyst, was then added dropwise through the funnel to initiate the hydrosilylation reaction. The catalyst dosage was precisely controlled to prevent over-crosslinking or premature gelation. Given the exothermic nature of the reaction, crushed ice was periodically added to the bath to control the temperature and prevent local overheating, which could otherwise affect the reaction rate and molecular architecture.

The reaction was allowed to proceed under continuous stirring for 8 to 12 h to ensure complete conversion of reactive groups. Upon completion, the resulting product was a viscous, transparent liquid precursor with excellent flowability.

### 2.3. Characterization

The pyrolysis behavior and thermal stability of PVSO were evaluated under an air atmosphere using a simultaneous thermal analyzer (SDT650, TA Instruments, New Castle, DE, USA), operated in TG-DSC mode at a heating rate of 10 °C/min from room temperature to 1000 °C, with an alumina crucible and a sample mass of approximately 10 mg. The structural evolution of PVSO during pyrolysis at different temperatures (400–1000 °C) was investigated by Fourier-Transform Infrared Spectroscopy (FT-IR, Nicolet 5700, Thermo Fisher Scientific, Waltham, MA, USA) in the range of 400–4000 cm^−1^ using the KBr pellet method with a resolution of 4 cm^−1^ and 32 scans per spectrum. The molecular structure of the precursor was further characterized by ^1^H and ^29^Si Nuclear Magnetic Resonance (NMR) spectroscopy (AVANCE III HD 400 MHz, Bruker, Berlin, Germany) using CDCl_3_ as the solvent and tetramethylsilane (TMS) as the internal standard. Phase compositions of the pyrolyzed samples were identified by X-ray diffraction (XRD, Rigaku D/MAX-2500, Akishima, Tokyo, Japan), scanned in the 2θ range of 10–80° at a scanning rate of 5°/min with Cu Kα radiation (λ = 0.15406 nm). Dielectric properties of the ceramic products were measured in the X-band frequency range (8.2–12.4 GHz) using a waveguide method with a vector network analyzer (MS4644A, Anritsu, Atsugi, Japan); the samples were machined into standard rectangular waveguide dimensions for accurate permittivity and loss tangent determination.

To evaluate the curing behavior of PVSO, non-isothermal differential scanning calorimetry (DSC) measurements were performed at multiple heating rates (*β* = 5, 10, 15, and 20 °C/min). The kinetic parameters of the curing reaction, including the activation energy (*E_a_*), were calculated using the *Kissinger* and *Ozawa* models. The *Kissinger* equation is given by the following:

ln(*β*/*T_p_*^2^) = −*E_a_*/*(R·T_p_*) + constant (1)

where *β* is the heating rate, *T_p_* is the peak curing temperature, *E_a_* is the apparent activation energy, and *R* is the universal gas constant (8.314 J/mol·K).

For comparison, the *Ozawa* method was also employed, which does not require knowledge of the reaction order and is expressed as follows:

ln(*β*) = −1.052(*E_a_*/*R*)(1/*T_p_*) + constant (2)

These equations were used to analyze the DSC data and determine the kinetic parameters associated with the curing behavior of PVSO, as detailed in Section 3.7.

## 3. Results and Discussion

### 3.1. Mechanism of PVSO Synthesis

PVSO was synthesized via a ring-opening polymerization reaction between D_4_^H^ and D_4_^Vi^ under catalytic conditions. TMAH was employed as an effective base catalyst to initiate the polymerization. The ring-opening polymerization of cyclic siloxanes proceeded through a reversible equilibrium mechanism, in which TMAH played a dual role—facilitating both the ring-opening polymerization and potential back-reaction via depolymerization. Consequently, to suppress depolymerization and ensure the stability of the resulting polymer, it was necessary to remove the catalyst at the later stages of the reaction. This requirement added complexity to the synthesis process, necessitating careful control over reaction conditions and catalyst removal procedures.

**Figure 1:**
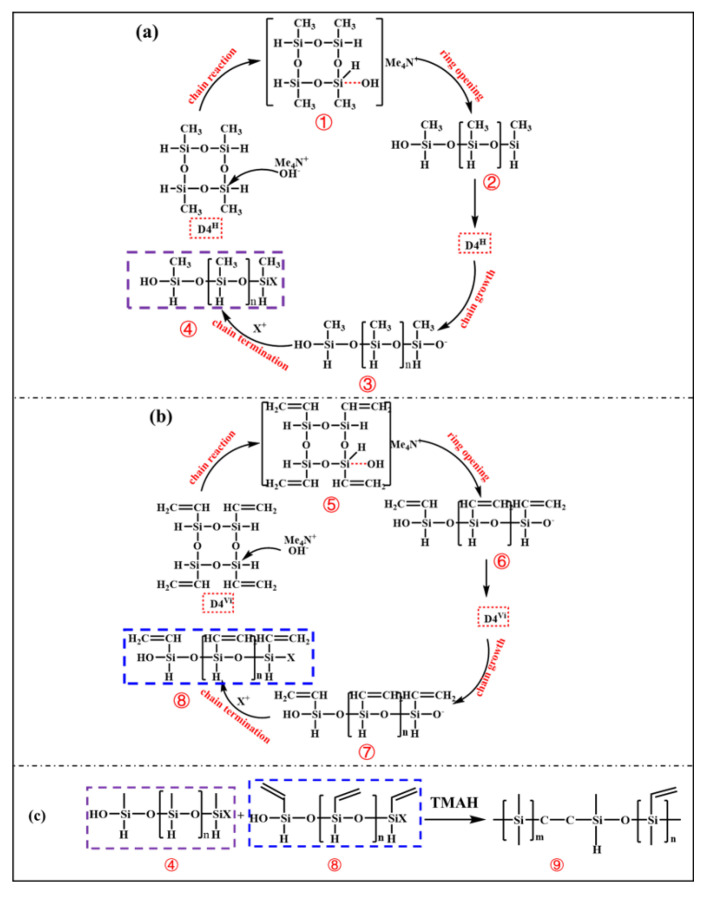
Synthesis of vinyl-containing polysiloxane: (**a**) ring-opening of D_4_^H^, (**b**) ring-opening of D_4_^Vi^, (**c**) hydrosilylation reaction.

Ideally, the synthesis of PVSO involves two sequential steps [23]: (1) ring-opening polymerization of D_4_^H^ and D_4_^Vi^ to yield reactive linear or branched siloxane chains; (2) hydrosilylation between Si-H and vinyl groups to construct a three-dimensional crosslinked PVSO network. However, under practical conditions, these two processes—polymerization and hydrosilylation—occur simultaneously upon ring-opening initiation. The proposed reaction mechanism was schematically illustrated in Figure 1. Under thermal or catalytic conditions, the cyclic siloxane monomers D_4_^H^ and D_4_^Vi^ undergo ring-opening polymerization to generate reactive intermediates bearing terminal functional groups. In the initiation stage, TMAH serves as a strong base catalyst. The hydroxide anion (OH^−^) coordinates with the silicon atoms in D_4_^H^ and D_4_^Vi^ via interaction with the vacant 3d orbitals of silicon, thereby redistributing electron density within the siloxane ring [24]. This interaction increases the polarity of the -Si-O- bonds and weakens the -Si-O-Si-linkages, facilitating ring-opening cleavage at specific positions (① and ⑤). During the propagation stage, the resulting anion-terminated linear siloxane oligomers (② and ⑥) continue to react with additional D_4_^H^ and D_4_^Vi^ monomers, leading to chain growth and the formation of extended oligomers (③ and ⑦). With continued progression, these oligomers undergo successive ring-opening additions to yield high molecular weight linear polysiloxanes (④ and ⑧).

In the presence of TMAH, the -C=C- in the vinyl groups was activated. The amino functionality (-NH_2_) in TMAH exhibited notable nucleophilicity and interacted with the electron-deficient carbon atoms of the vinyl groups, thereby increasing their electrophilic character and facilitating the subsequent hydrosilylation reaction [25]. During this process, the Si-H moiety underwent addition across the activated double bonds. The hydrogen atom from the Si-H bond attached to one carbon of the vinyl group, while the silicon atom formed a covalent bond with the adjacent carbon, resulting in the formation of a -Si-CH_2_-CH_2_-Si- linkage. This hydrosilylation constituted a chain-growth addition mechanism, wherein successive additions between Si-H and vinyl groups progressively increased the molecular weight of the polymer [26]. Continued crosslinking ultimately yielded a three-dimensional network structure, corresponding to the final crosslinked polysiloxane species (⑨).

### 3.2. Rheological Properties of PVSO

The viscosity of the PVSO precursor was measured to be in the range of 45–60 mPa·s at 25 °C, exhibiting Newtonian flow behavior across a shear rate range of 0.1–100 s^−1^. The stable and moderate viscosity is favorable for effective infiltration into porous preforms, while still ensuring sufficient wettability and process control.

### 3.3. FT-IR Analysis of PVSO

**Figure 2:**
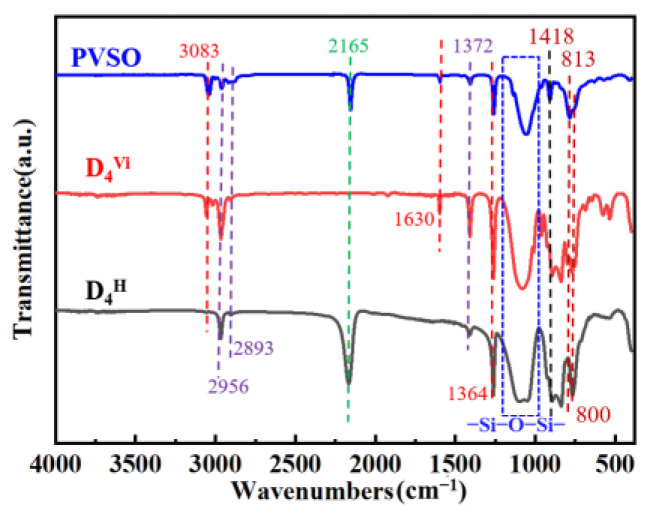
The FT-IR spectrum of raw materials and products(PVSO).

The ideal PVSO product comprises a three-dimensional network structure characterized by alternating siloxane linkages (-Si-O-Si-) and -Si-C-C-Si- bonds. The polymer-sided chains contain critical functional groups, including unsaturated -C=C-, --Si-CH_3_-, and -Si-H. FT-IR spectroscopy of the synthesized PVSO, shown in Figure 2, confirms the presence of these characteristic moieties [27]. The FT-IR spectrum of the synthesized PVSO product exhibited several characteristic absorption bands in Figure 2. The peak at 3083 cm^−1^ was attributed to the stretching vibration of =C-H bonds, while the peaks at 2956 cm^−1^ and 2893 cm^−1^ corresponded to the asymmetric and symmetric stretching vibrations of -CH_2_- groups, respectively. The absorption at 1630 cm^−1^ was assigned to the stretching vibration of -C=C-, indicating incomplete consumption of vinyl groups during the reaction and the presence of residual unsaturation. The bands at 1364 cm^−1^ and 1418 cm^−1^ arose from the symmetric deformation vibration of -CH_2_- and the asymmetric stretching vibration of -C-H, respectively, confirming the existence of -CH_2_-CH_2_- linkages formed by hydrosilylation [28]. A strong absorption between 1000 cm^−1^ and 1250 cm^−1^ corresponded to the asymmetric stretching of -Si-O-Si- bonds, characteristic of the siloxane backbone structure. Peaks at 800 cm^−1^ and 813 cm^−1^ were assigned to the symmetric and asymmetric bending vibrations of dimethyl groups attached to -Si(CH_3_)_2_-, further verifying the incorporation of these substituents. Notably, a diminished peak near 2165 cm^−1^, attributed to -Si-H stretching vibrations, indicated partial consumption of Si-H bonds during hydrosilylation. The concurrent reduction in -C=C- and -Si-H peak intensities, along with the emergence of characteristic -C-C- single bond absorptions, provided strong evidence for successful hydrosilylation between D_4_^H^ and D_4_^Vi^, resulting in the formation of a crosslinked PVSO network

### 3.4. Nuclear Magnetic Resonance (NMR) Analysis of PVSO

**Figure 3:**
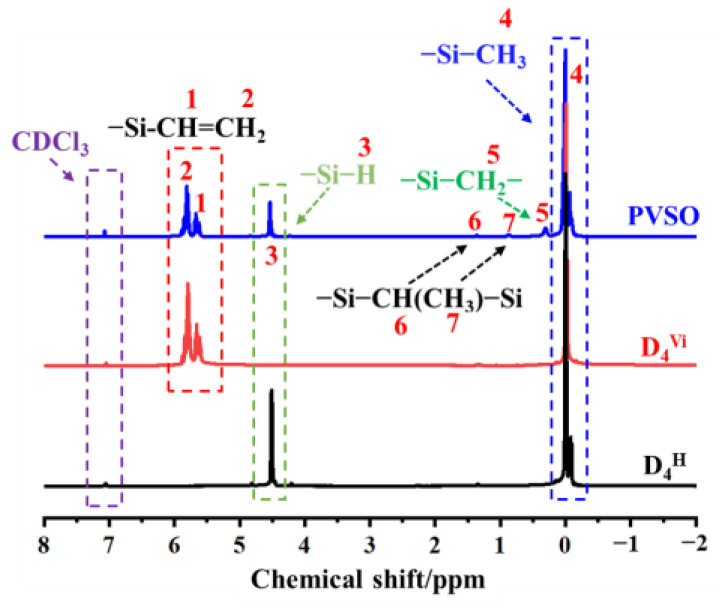
The ^1^H-NMR spectra of PVSO.

To further elucidate the molecular architecture of PVSO, ^1^H-NMR spectroscopy was conducted, with the spectrum presented in Figure 3. Comparison of the ^1^H-NMR spectra of the product and the starting materials revealed a marked attenuation of the characteristic peak at δ = 4.67 ppm, attributed to the -Si-H protons, indicating consumption of this functional group during the reaction. Concurrently, a new resonance emerged at δ = 0.41 ppm, corresponding to -Si-CH_2_- formed via hydrosilylation between -Si-CH=CH_2_ and -Si-H groups, confirming successful crosslinking through Si-H addition across vinyl groups. The methyl protons attached to -Si-CH_3_ give rise to a signal near δ = 0 ppm [29]. Vinyl protons on the first and second carbon atoms of the -Si-CH=CH_2_ moiety appear at δ = 5.82 ppm and δ = 5.96 ppm, respectively. Additionally, due to distinct binding modes between -Si-CH=CH_2_ and -Si-H, a minor presence of -Si-CH(CH_3_)-Si- groups was detected, with methyl protons resonating at δ = 1.13 ppm. Collectively, these ^1^H-NMR results provide compelling evidence for the occurrence of hydrosilylation and the formation of the targeted three-dimensional crosslinked PVSO network.

**Figure 4:**
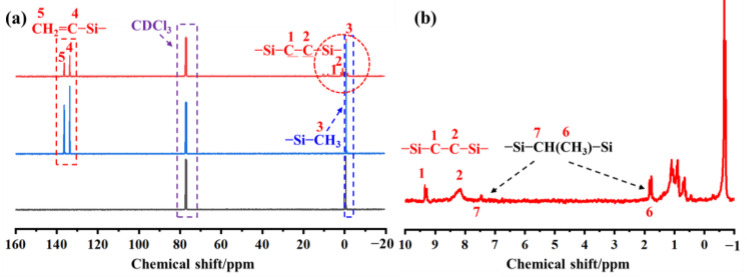
The Nuclear Magnetic Resonance (NMR) spectra of PVSO: (**a**) ^13^C-NMR spectrum; (**b**) Magnified view of −1~10 ppm.

Figure 4a displays the ^13^C-NMR spectrum of PVSO, with an expanded view of the chemical shifts between −1 and 10 ppm shown in Figure 4b. The corresponding chemical shift assignments are detailed in Table 1. In Figure 4a, five peaks observed in the δ = −1 to 1 ppm region are attributed to four distinct chemical environments of the -Si-CH_3_ groups. Resonances at δ = 8.25 ppm and 9.37 ppm corresponded to methylene carbons within the -Si-CH_2_-CH_2_-Si- segments. Peaks at δ = 134.21 ppm and 137.55 ppm were assigned to the vinyl carbons (first and second carbons, respectively) of the -Si-CH=CH_2_ groups [30]. Furthermore, the expanded spectrum (Figure 4b) revealed a chemical shift near δ = 7.82 ppm, which is attributed to the carbon atom in the -Si-CH(CH_3_)-Si- moiety.

### 3.5. Thermosetting Mechanism of PVSO

**Figure 5:**
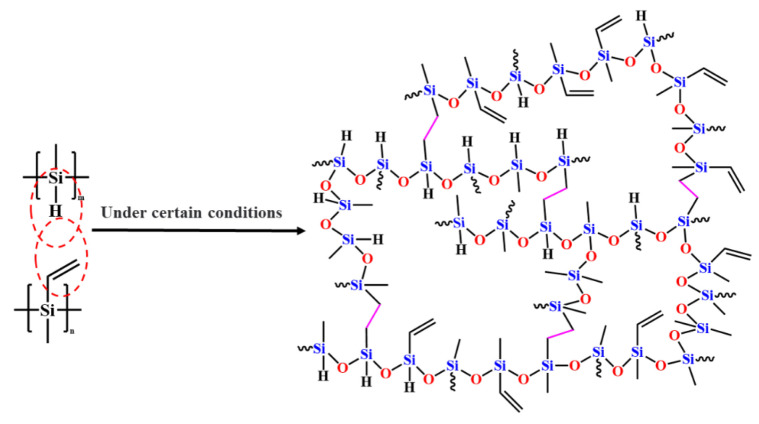
Curing mechanism of PVSO.

The thermosetting behavior of PVSO primarily originated from the incomplete hydrosilylation reaction during precursor synthesis, which left residual reactive groups such as -Si-H and -C=C- within the polymer network. Upon heating during the curing process, the increased thermal energy facilitated further reactions between these residual functional groups [31]. Once the curing temperature was reached, these active groups undergo additional crosslinking through hydrosilylation, wherein silane hydride groups reacted with carbon-carbon double bonds to form new -Si-C-C-Si- linkages, concomitantly converting vinyl double bonds into carbon-carbon single bonds, as schematically illustrated in Figure 5. Progression of curing led to the consumption of residual vinyl and silane hydride groups, resulting in a more densely crosslinked and robust molecular network. This enhanced crosslink density was critical for improving the ceramic yield during subsequent pyrolysis.

### 3.6. Non-Isothermal DSC Analysis of PVSO

**Figure 6:**
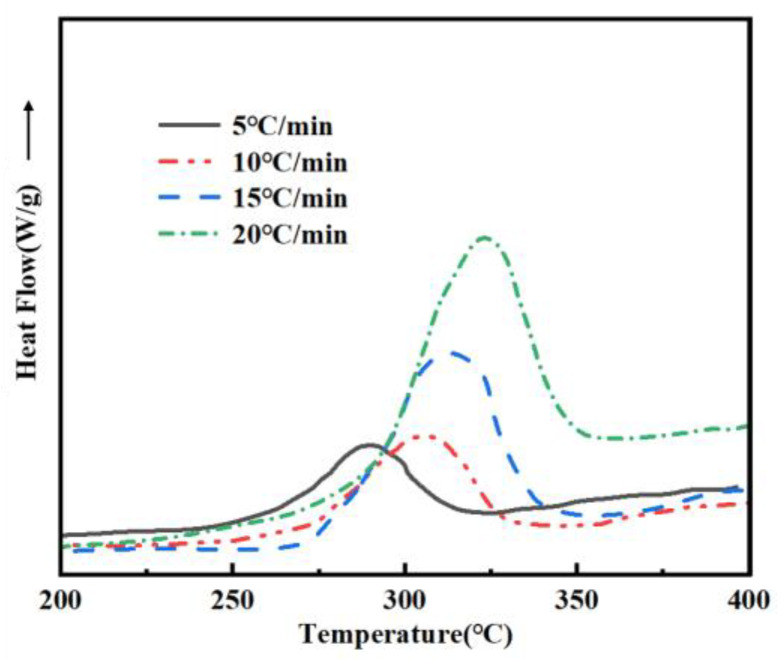
DSC curves of PVSO at different *β* values.

To elucidate the curing mechanism of PVSO, DSC measurements were conducted at multiple heating rates (*β* = 5, 10, 15, and 20 °C/min). The DSC curves obtained under these conditions were analyzed to extract the initial curing temperature *T_i_*, peak curing temperature *T_p_*, and final curing temperature *T_f_* of PVSO. Subsequently, the *T_p_* were employed to calculate the activation energy *E_a_* of the curing reaction using appropriate kinetic models. This approach facilitated a detailed understanding of the curing kinetics and enabled a comprehensive interpretation of the non-isothermal curing behavior of PVSO. Figure 6 presents the DSC curves of PVSO recorded at various heating rates, illustrating its exothermic and endothermic behavior as a function of temperature. With increasing heating rates, the exothermic peaks became sharper and the temperature range over which the reaction occurred narrowed. Moreover, the peak temperatures shift systematically towards higher values [32]. This behavior was attributed to the reduced reaction time at elevated heating rates, which required a higher temperature to achieve an equivalent degree of curing. In contrast, slower heating rates allowed the reaction to proceed over a longer duration at a given temperature, resulting in exothermic peaks at relatively lower temperatures for the same conversion extent.

**Figure 7:**
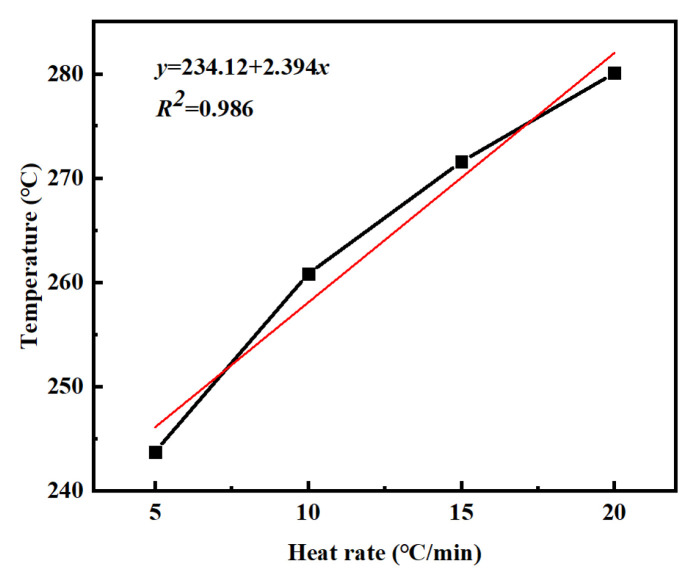
*T_i_-β* curves of PVSO at different heating rates.

Table 2 summarizes the *T_i_*, *T_p_*, and *T_f_* of PVSO measured at different heating rates *β*. Linear regression analyses were performed by plotting *β* as the independent variable against *T_i_*, *T_p_*, and *T_f_*, respectively. The resulting linear fits, obtained via the least squares method, accurately described the dependence of these characteristic curing temperatures on the heating rate, providing valuable insights into the curing kinetics of PVSO. Figure 7 illustrates the relationship between the reaction onset temperature *T_i_* of PVSO and the heating rate *β*. Linear fitting of the experimental data yields the curing reaction equation [33].

y = 234.12 + 2.394x
(3)

**Figure 8:**
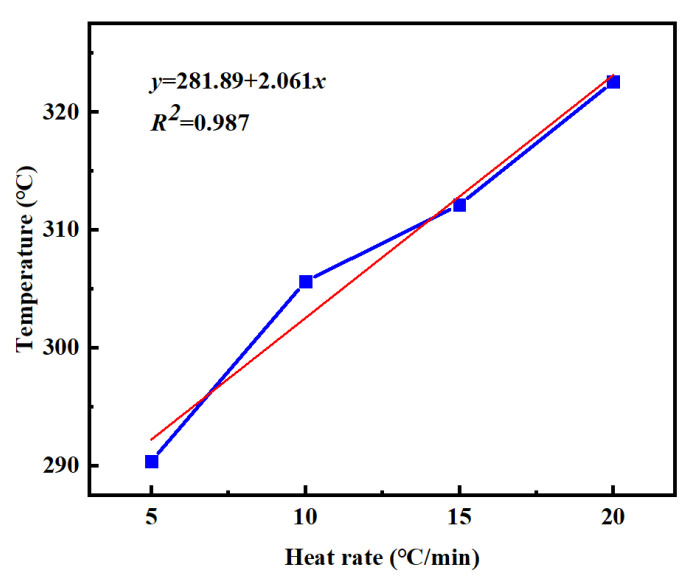
*T_p_*-*β* curves of PVSO at different heating rates.

From this equation, the minimum curing temperature of PVSO is determined to be 234.12 °C. Figure 8 shows the correlation between the peak curing temperature *T_p_* of PVSO and the heating rate *β*. Linear regression of the data yields the following curing reaction equation [34].

y = 281.89 + 2.061x
(4)

**Figure 9:**
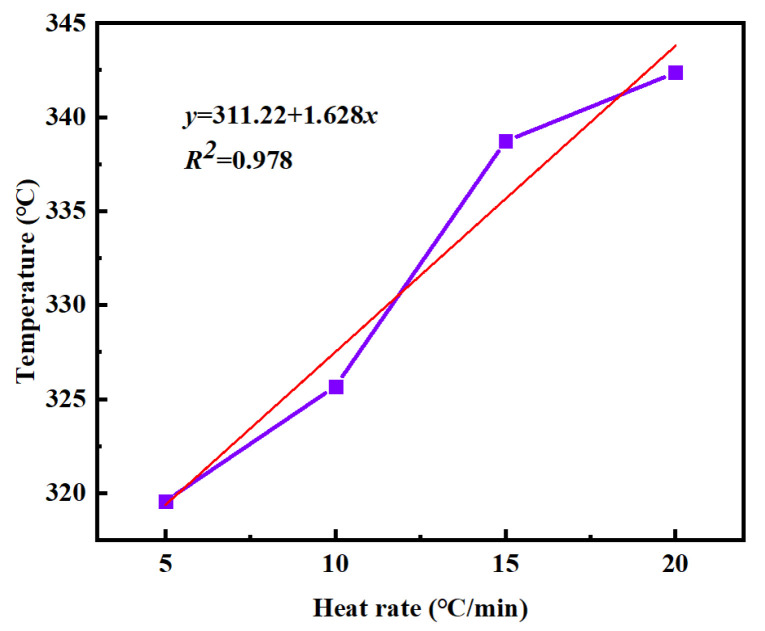
*T_f_*-*β* curves of PVSO at different heating rates.

Based on this equation, the maximum curing temperature of PVSO is determined to be 281.89 °C. Figure 9 shows the relationship between the reaction termination temperature *T_f_* of PVSO and the heating rate *β*. Linear fitting yields the curing reaction equation [35].

y = 311.22 + 1.628xy
(5)

From this equation, the termination temperature of the PVSO curing reaction is determined to be 311.22 °C. In summary, the characteristic temperatures of the static curing reaction of PVSO were determined as follows: the initial curing temperature *T_i_* = 234.12 °C, the peak curing temperature *T_p_* = 281.89 °C, and the termination curing temperature *T_f_* = 311.22 °C. By extrapolating the linear relationships between these temperatures and the heating rate to a heating rate of zero, theoretical key temperature points for the curing process, namely the initial curing temperature, main curing temperature, and post-curing temperature, were obtained [36]. These values provided a theoretical foundation for the design of stepwise curing protocols. The static curing parameters are summarized in Table 3. The high correlation coefficients (*R*^2^ > 0.95) for all three fitted equations indicate a strong linear dependence of the characteristic curing temperatures *T_i_*, *T_p_*, and *T_f_* on the heating rate *β*, confirming that the curing reaction temperature was heating rate-dependent.

### 3.7. Curing Kinetics of PESO

The apparent activation energy *E_a_* is a key kinetic parameter for characterizing the curing behavior of thermosetting resin systems. Changes in *E_a_* often reflect variations in the curing mechanism and can be used to interpret the resin’s non-isothermal curing behavior. Among the various evaluation methods, the Kissinger approach is widely recognized as an effective and reliable technique. This method assumes that the curing reaction follows an nth-order kinetic model, where the total reaction enthalpy is proportional to the conversion degree *α*, and the temperature dependence of the reaction rate constant (*k*(*T*)) obeys the Arrhenius equation. Under these assumptions, the reaction rate can be expressed as follows [37]:

(6) $$\frac{d\alpha }{dt}=\frac{\frac{dH}{dt}}{\Delta H_{0}}=k(T)f(\alpha )=A\exp(\frac{-E_{a}}{RT})f(\alpha )$$

where *α* represents the degree of conversion; *dα*/*dt* denotes the reaction rate; *dH*/*dt* is the heat flow rate of the reaction; Δ*H* is the total reaction enthalpy; *f*(*α*) is the kinetic model function; *A* is the pre-exponential factor; and *R* is the universal gas constant, typically taken as 8.314 J/(mol·K).

The apparent activation energy *E_a_* of the resin curing system can be calculated using the Kissinger equation, as expressed in Equation (7) [38]:

(7) $$\ln\left(\frac{\beta }{{T_{p}}^{2}}\right)=\ln\left(\frac{A\times R}{E_{a}}\right)-\frac{E_{a}}{R\times T_{p}}$$

From the above equation, the following can be derived [39]:

(8) $$\frac{d [\ln\left(\frac{\beta }{{T_{p}}^{2}}\right)]}{d\left(\frac{1}{T_{p}}\right)}=\frac{-E_{a}}{R}$$

**Figure 10:**
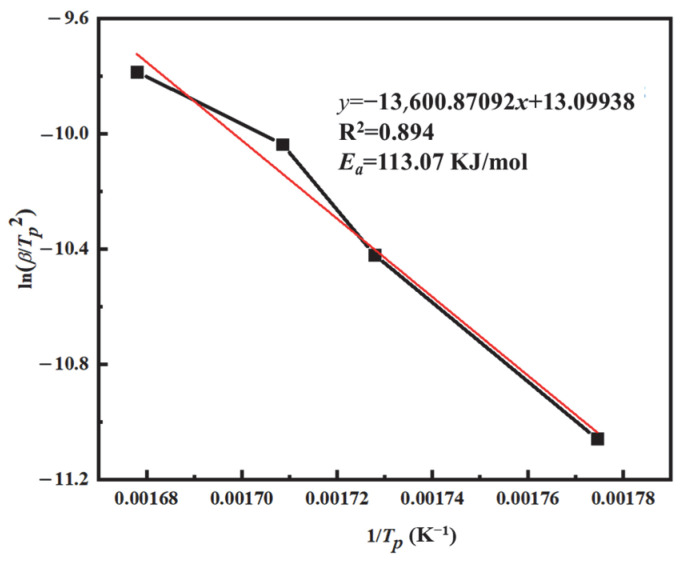
The ln(*β*/T_p_^2^) − 1/*T_p_* curve of PVSO.

Figure 10 presents the plot of ln*β/T_P_*^2^ versus 1/*T_P_* for the PVSO resin curing system. Based on the linear fitting of the curve, the slope of the fitted line was used to calculate the apparent activation energy *E_a_*, which was determined to be 113.07 kJ/mol. The apparent activation energy *E_a_* can also be evaluated using the Ozawa method, which is analogous to the Kissinger approach but does not require knowledge of the reaction order *n*. As shown in Equation (6), this characteristic makes the Ozawa method particularly advantageous, as it reduces uncertainties related to reaction mechanisms and directly provides the apparent activation energy. The Ozawa equation is expressed as follows [40]:

(9) $$\ln\beta =\ln\left(\frac{AE_{a}}{R}\right)-\ln F(\alpha )-5.3331-1.052(\frac{E_{a}}{RT_{p}})$$

**Figure 11:**
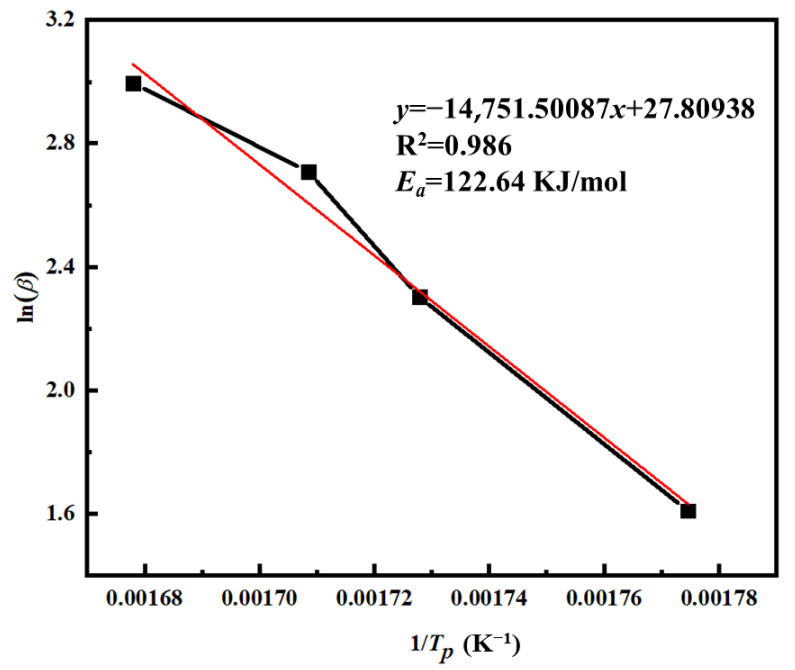
The ln(*β*) − 1/*T_p_* curve of PVSO.

In this equation, *F*(*α*) is considered a constant function. Based on the Ozawa equation, a linear fitting was performed using ln*β* as the dependent variable and 1/*T_p_* as the independent variable, as shown in Figure 11. The resulting regression exhibited a good linear correlation with an *R*^2^ value exceeding 0.986. From the slope of the fitted line, the apparent activation energy *E_a_* was calculated to be 122.64 kJ/mol.

A comparison of the activation energies determined via the Ozawa and Kissinger methods revealed close agreement between the two approaches. Minor discrepancies arise primarily from differences in the underlying assumptions of each method. The Kissinger method relied on the temperature at the maximum reaction rate and assumed that the influence of the reaction order was negligible throughout the curing process. Conversely, the Ozawa method analyzed conversion data under non-isothermal conditions at multiple heating rates, thus capturing the staged nature of the reaction more explicitly. These distinctions in data treatment and kinetic assumptions may result in slight variations in the calculated activation energies [41]. Nonetheless, the close correspondence between the values indicated the robustness of the chosen kinetic models. This consistency further supports that the nth-order reaction model effectively described the curing kinetics of PVSO, providing an accurate representation of its polymerization behavior.

The activation energies derived from the Ozawa and Kissinger methods exhibit close agreement, with only slight deviations attributed to the differing assumptions underlying each approach. This consistency further validated the suitability of the nth-order reaction model in accurately describing the curing kinetics of PVSO [42].

To minimize errors arising from different methods used to calculate the activation energy *E_a_*, the average value *E_a_* obtained by the Ozawa and Kissinger methods can be taken [43].

(113.07 + 122.64)/2 = 117.855 KJ/mol
(10)

For subsequent calculations of the pre-exponential factor *A*, by substituting *E_a_* = 117.855 kJ/mol into the Kissinger equation (Equation (7)) and using the intercept from the linear fit, the pre-exponential factor *A* is calculated to be 6.86 × 10^9^ s^−1^.

The reaction order *n* characterizes the reaction mechanism and can, to some extent, reflect the complexity of the curing reaction. By rearranging Equation (10) and applying a logarithmic transformation, followed by differentiation with respect to 1/*T_p_*, the following expression, Equation (11), is obtained [44].

(11) $$\frac{d(\ln\beta )}{d(\frac{1}{T_{p}})}= [\frac{(n-1){T_{p}}^{2}}{1-\alpha_{p}}]\frac{d\alpha }{\beta dt}-2T_{p}-\frac{E_{a}}{R}$$

The kinetic model function for an n-th order reaction is expressed as follows [45]:

(12) $$f(\alpha )={\left(1-\alpha \right)}^{n}$$

By substituting the rate constant equation and the curing kinetic function into the kinetic equation, the curing model equation can be derived as follows [46]:

(13) $$\frac{d\alpha }{dt}=k(1-\alpha )=A\exp(-\frac{E_{a}}{RT}){\left(1-\alpha \right)}^{n}$$

(14) $$\frac{d\alpha }{\beta dt}=\frac{1-\alpha_{p}}{n}\frac{E_{a}}{R{T_{p}}^{2}}$$

By substituting Equation (14) into Equation (9), the Crane equation can be derived as follows [47]:

(15) $$\frac{d(\ln\beta )}{d(1/T_{p})}=-(\frac{E_{a}}{nR}+2T_{p})$$

In the equation, *n* represents the reaction order. Equation (15) can be simplified as follows [48]:

(16) $$\frac{d(\ln\beta )}{d(1/T_{p})}=-\frac{E_{a}}{nR}$$

In the equation, *β* is the heating rate (K/s), *T_p_* is the peak temperature (K), *A* is the pre-exponential factor, *R* is the gas constant, generally taken as 8.314 J/(mol·K), *E_a_* is the apparent activation energy of the curing reaction (J/mol); and *n* is the reaction order.

In this study, 2*T_p_* ≈ 1.11 × 10^3^ K. Therefore, an approximate calculation can be performed based on Equation (10). From the slope of the fitted line, the reaction order nnn was determined to be 0.96, with an *R*^2^ value greater than 0.98, indicating a strong linear correlation. Substituting the obtained kinetic parameters-the pre-exponential factor *A* = 6.86 × 10^9^ s^−1^, *n* = 0.960, *E_a_* = 117.855 kJ/mol into Equation (17) yields [49].

(17) $$\frac{d\alpha }{dt}=k(1-\alpha )=6.86\times 10^{9}\exp(-\frac{13,600}{T+273.15}){\left(1-\alpha \right)}^{0.96}$$

**Figure 12:**
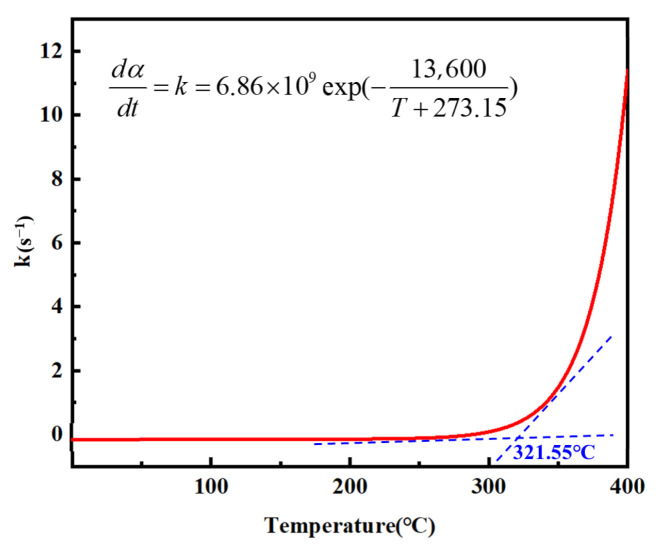
*n*-order curing reaction equation curve.

Before the onset of the curing reaction, the degree of cure *α* of the resin is zero. By substituting *α* = 0 into the curing kinetics equation Equation (15), the relationship between the initial curing rate and temperature can be derived, from which the curing rate-temperature curve shown in Figure 12 was constructed. As illustrated, at low temperatures, the system lacks sufficient thermal energy to overcome the activation barrier, resulting in an essentially negligible curing rate. Upon gradual temperature increase, the reaction activity was thermally activated, leading to a sharp rise in the curing rate.

**Figure 13:**
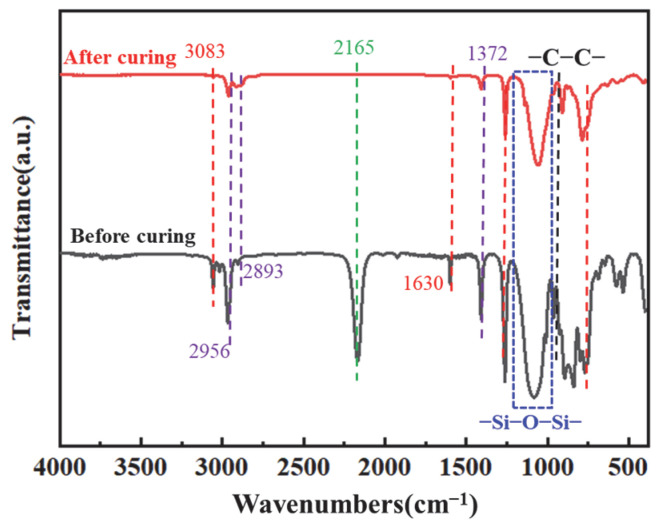
FT-IR spectrum of PVSO.

The curing kinetics of the PVSO resin system were systematically investigated and integrated with the theoretical curing process to optimize the curing treatment and maximize the degree of cure. Figure 13 presents the FT-IR spectra of the samples before and after curing, illustrating the chemical changes associated with the curing reaction. The FT-IR spectra in Figure 13 reveal notable changes following curing. The characteristic =C-H stretching vibration at 3083 cm^−1^, along with the asymmetric and symmetric -CH_2_- stretching vibrations at 2956 cm^−1^ and 2893 cm^−1^, respectively, exhibit significant attenuation, indicating a decrease in C-H bond content concurrent with the increasing crosslink density [50]. This reduction was attributed to the gradual formation of the PVSO crosslinked network, during which some methylene side groups were lost as volatile small molecules, resulting in an overall decrease in carbon content. Using the relatively stable -Si-O-Si- absorption peak as an internal reference, the areas corresponding to the -C=C- stretching vibration at 1630 cm^−1^ and the -Si-H bond absorption at 2165 cm^−1^ markedly diminish, confirming the consumption of these reactive groups via hydrosilylation reactions during curing. This process led to enhanced crosslink density, which in turn improved the cured product’s thermal stability and mechanical strength.

### 3.8. Polymer-to-Ceramic Conversion Process of PESO

**Figure 14:**
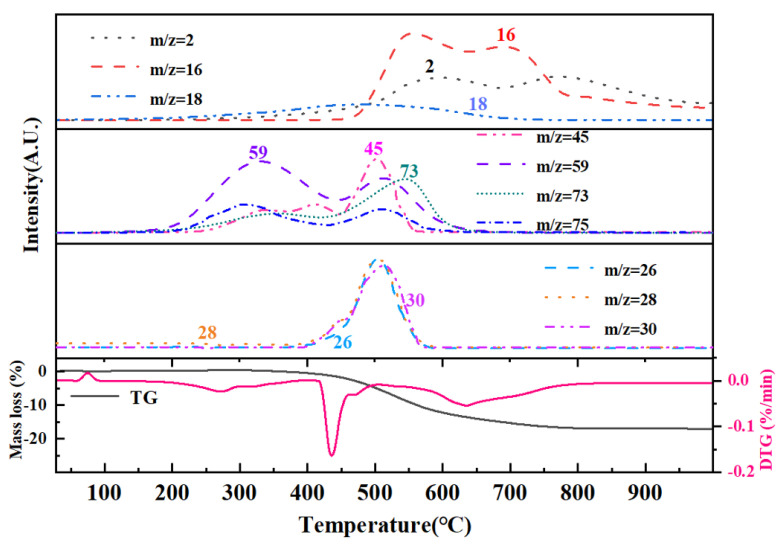
The TG-MS curve of PVSO.

Thermal pyrolysis constituted the final and pivotal step in the polymer-to-ceramic conversion process, exerting a profound influence on the microstructural evolution of the resultant ceramic. The pyrolysis behavior of ceramic precursors was inherently complex, particularly within the 400–800 °C temperature range, where intense reactions and multiple concurrent mechanisms take place. During this stage, numerous reactive organic moieties-including methyl and vinyl groups-and chemical bonds such as -Si-OH, -Si-H, and -Si-NH_x_ progressively decompose and vanish. This decomposition was accompanied by free radical generation and subsequent condensation crosslinking reactions. Concurrently, the material experiences pronounced volume shrinkage, triggering densification and rapid molecular rearrangement of the precursor’s structure into an inorganic network, thereby forming the nascent ceramic framework. This critical transition window governs the evolution from polymeric precursor to ceramic, decisively impacting the final phase composition, microstructure, and properties of the ceramic product [51]. TGA provided insights into the mass loss profile during pyrolysis, elucidating the decomposition behavior across different temperatures and thereby guiding process optimization. The TG-MS coupled thermal analysis of PVSO is presented in Figure 14, with Table 4 summarizing the principal gaseous species evolved during the pyrolysis of liquid PVSO. Owing to the complexity of fragmentation, only the most significant ions were highlighted to clearly represent the MS spectra.

The first stage occurred between room temperature and 400 °C, characterized by a slight weight loss primarily due to the volatilization of small molecules from incompletely crosslinked regions within the PVSO matrix. Given the relatively low concentration of -Si-OH groups, hydrolysis and subsequent H_2_O elimination are minimal. The evolved gases detected at this stage mainly include H_2_O (*m*/*z* = 18) and silicon-containing fragments, predominantly(CH_3_)_3_Si-(*m*/*z* = 59, 73), which correspond to the terminal groups of the vinyl polysiloxane polymer. The presence of H_2_O facilitated hydrolysis at these terminal sites and the decomposition of (CH_3_)_3_Si-OH (*m*/*z* = 75, 45), as illustrated in Reactions (18) and (19) [52].




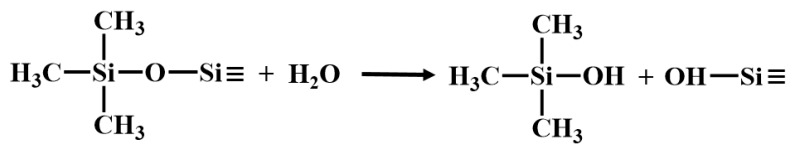

(18)






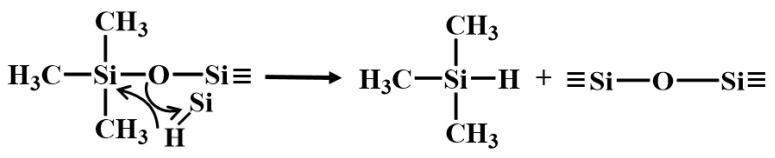

(19)



The second stage occurred between 400 °C and 530 °C, during which PVSO experienced further mass loss primarily due to continued curing and crosslinking. This stage was dominated by hydrosilylation reactions that promoted the formation of a three-dimensional network structure. Correspondingly, a range of silicon-containing fragments were detected, initially including (CH_3_)SiH_2_- (*m*/*z* = 45, 44, 43, 31, 30), followed by (CH_3_)_2_SiH- (*m*/*z* = 59, 58, 45, 44, 43) and (CH_3_)_3_Si- (*m*/*z* = 73, 59, 42). Additionally, cleavage of Si-H bonds generated hydrogen radicals that recombine with carbon-centered radicals, leading to the evolution of small alkyl hydrides such as methane CH_4_ (*m*/*z* = 16, 15, 14, 12), ethylene C_2_H_4_ (*m*/*z* = 28, 30), and ethane C_2_H_6_ (*m*/*z* = 28, 26) [53].

The third stage occurred within the temperature range of 530 °C to 750 °C, during which inorganic reactions proceed further. The mass loss at this stage was primarily attributed to the cleavage of CH_2_ and CH_3_ groups, accompanied by the release of small molecules such as H_2_ (*m*/*z* = 2) and CH_4_. The pronounced CH_4_ peak observed around 550 °C likely arose from the cleavage of -Si-CH_3_ bonds or -CH_2_-CH_2_- linkages within the polymer network. The bond dissociation energies in this system were as follows: Si-O (531–812 kJ/mol), C-H (411 kJ/mol), Si-H (339 kJ/mol), C-C (334 kJ/mol), and Si-C (306 kJ/mol). The comparatively high bond energy of Si-O bonds renders them more resistant to thermal cleavage, allowing Si and O atoms to remain more stably retained. Conversely, C and H atoms were preferentially released as small molecular fragments due to the cleavage of weaker bonds. Based on these considerations, the likely molecular chain scission reactions include the following [54]:

(20) $$\equiv Si-CH_{3}\to \equiv Si^{•}+C{H_{3}}^{•}$$

(21) $$\equiv Si-CH_{2}-CH_{2}-Si\equiv \to^{•}CH_{2}-Si\equiv$$

(22) $$\equiv Si-CH_{2}-\to \equiv Si^{•}{+}^{•}CH_{2}-$$

(23) $$\equiv Si-H\to \equiv Si^{•}{+}^{•}H$$

(24) $$\equiv C-H-\to \equiv C^{•}+H^{•}$$

The fourth stage occurred between 750 °C and 1000 °C, during which PVSO exhibited minimal mass loss, indicating that the precursor’s weight remained essentially stable. This suggested that at approximately 750 °C, the organic to inorganic transformation was essentially complete, with the precursor successfully converting into silica ceramic. The inorganic reaction had largely concluded, marking the termination of the pyrolysis process. The final ceramic yield reached 81.3%, which was highly advantageous for the fabrication of fiber-reinforced ceramic matrix composites via the polymer infiltration and pyrolysis (PIP) method.

**Figure 15:**
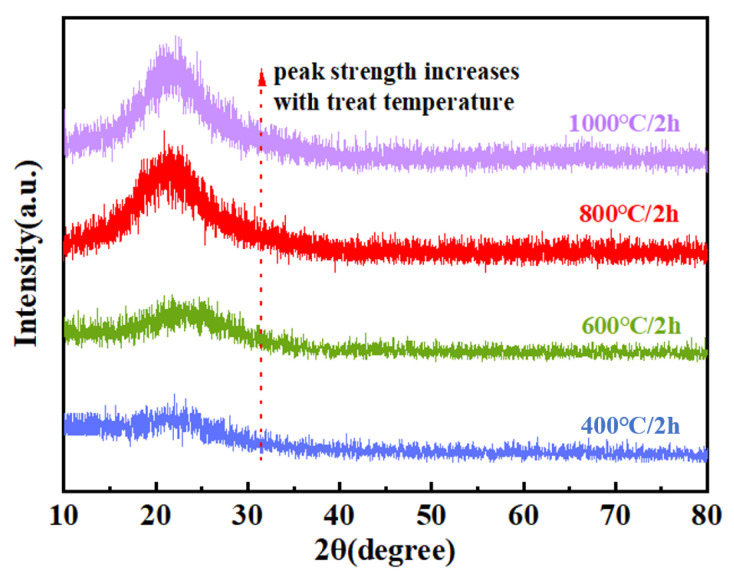
The XRD patterns of PVSO pyrolyzed at different temperatures.

The XRD analysis was performed to investigate the phase composition of the pyrolysis products obtained at different temperatures, with the corresponding diffraction patterns presented in Figure 15. The relative elemental compositions of the PVSO-derived silica ceramics at various pyrolysis temperatures are summarized in Table 5. The XRD analysis revealed that within the pyrolysis temperature range of 400–1000 °C, no distinct crystalline diffraction peaks were observed for PVSO; instead, broad diffuse halos dominated the patterns, indicating that the resulting silica ceramics primarily exhibited an amorphous structure. This suggests that during this stage of pyrolysis, the precursor had not yet developed an ordered crystalline framework and remains largely a disordered network. However, with increasing temperature, the intensity and sharpness of the characteristic diffraction peak near 22° gradually increased, reflecting enhanced structural ordering and a tendency towards crystallization, which became more pronounced above 600 °C [55]. Furthermore, the XRD patterns of the samples treated at 400 °C and 600 °C exhibited a subtle upward curvature at low diffraction angles, which disappeared upon heating to 800 °C. This feature became more evident with increasing polymerization degree, indicating the presence of residual, incompletely decomposed organic moieties below 600 °C. Above 800 °C, these organic groups were effectively eliminated, confirming the complete transformation of the material into inorganic silica ceramic.

Based on the elemental composition of PVSO at different pyrolysis temperatures, the resulting silica ceramics primarily consist of Si, C, H, and O, with Si and O being the dominant elements. The source of oxygen was multifaceted; it originated both from oxygen atoms inherently present in the raw materials during precursor synthesis, which remained stably incorporated within the polymer backbone, and from atmospheric oxygen interacting with the precursor during pyrolysis. Particularly under high-temperature pyrolysis in air, oxygen from the atmosphere facilitated oxidative decomposition of the precursor’s organic constituents, yielding inorganic products such as SiO_2_. As the pyrolysis temperature increased, the organic moieties within the polysiloxane chains, especially carbon-containing groups, underwent progressive cleavage into small volatile molecules, including CO, CO_2_, and CH_4_, resulting in a marked decrease in carbon content. Concurrently, the cleavage of Si-O bonds and silicon dehydrogenation reactions promoted the formation of stable inorganic silicon oxides. Carbon was more readily oxidized into gaseous species at elevated temperatures. Notably, when the pyrolysis temperature reaches 600 °C, the residual carbon content sharply decreases to 0.283%, indicating that most carbon had been volatilized or oxidized, with the remaining carbon predominantly existing as free carbon within the pyrolysis residue. The reduction in residual carbon was beneficial for enhancing the electromagnetic wave transparency of the material, as carbon tended to increase electromagnetic absorption or reflection, thereby elevating dielectric loss and detrimentally affecting electromagnetic performance [56]. Consequently, pyrolysis products with lower residual carbon content exhibit superior high-frequency electromagnetic wave transmission characteristics.

**Figure 16:**
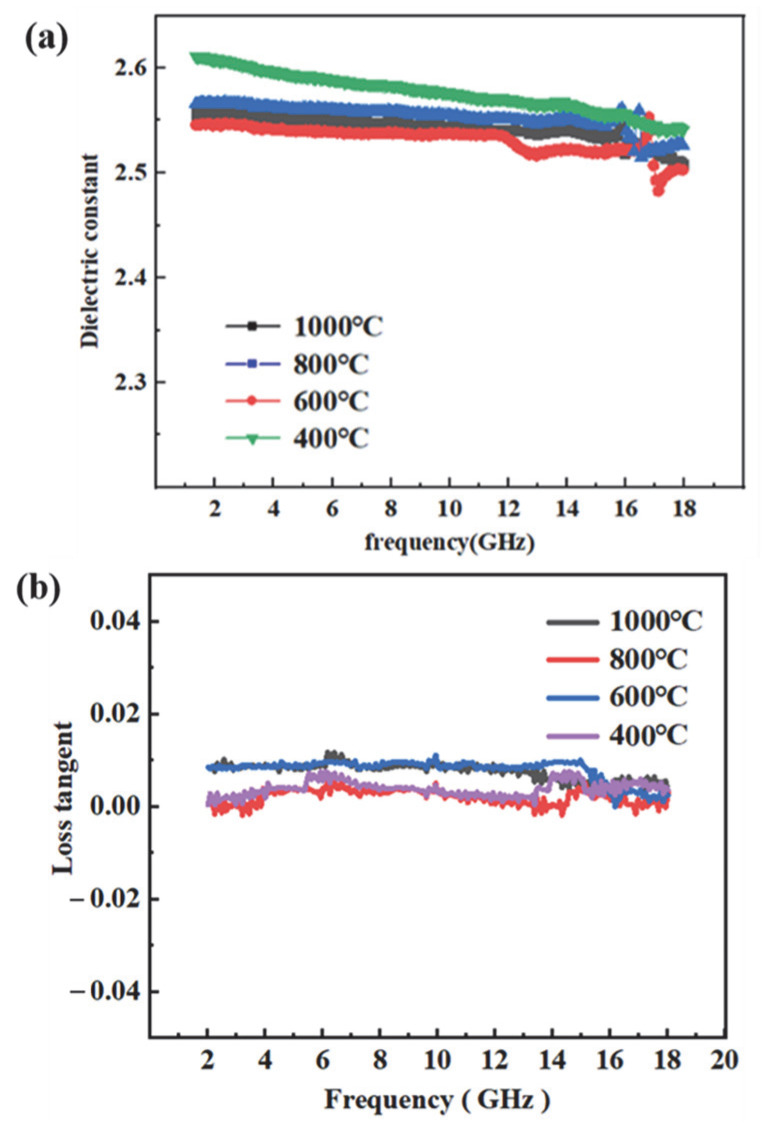
Dielectric properties of PVSO:(**a**) dielectric constant (ε); (**b**) dielectric loss tangent (tan δ).

As a critical protective component in radar systems, radomes must exhibit excellent electromagnetic wave transparency. The wave-transmitting performance of these materials was intimately linked to their dielectric properties; therefore, accurate measurement of the dielectric constant ε and dielectric loss tangent tan δ was essential to ensure optimal wave transmission. Figure 16. illustrated the variation in the dielectric constant of vinyl polysiloxane over the frequency range of 2–18 GHz. The data revealed that the ε remained relatively stable across this spectrum, showing no significant fluctuations. Moreover, an increase in temperature resulted in a slight decrease in the ε. This behavior was primarily attributed to the enhanced crosslinking within the precursor at elevated temperatures, which reduced the concentration of active polar groups. The resulting crosslinked network, characterized by lower polarity, led to a decreased ε. The dielectric response of vinyl polysiloxane can be interpreted in terms of the Debye equation (25), expressed as follows [57]:

(25) $$\frac{\left(k-1\right)}{\left(k+2\right)}=\frac{4\pi }{3}N\left(\alpha_{e}+\alpha_{d}+\frac{u^{2}}{3K_{b}T}\right)$$

In the equation, *k* represents the dielectric constant; *T* is the temperature; *N* denotes the dipole number density; *α_e_* and *α_d_* correspond to electronic and distortion polarizations, respectively; *u* is related to orientation polarization arising from dipole moments; and *K_b_* is the Boltzmann constant. According to the Debye equation, the dielectric constant is strongly influenced by the material’s polarizability and dipole density. Consequently, reducing either parameter resulted in a lower dielectric constant. Typical approaches to decreasing polarizability include incorporating chemical bonds with inherently low polarizability, such as C-F, C-H, and C-C bonds. Meanwhile, dipole density can be reduced by introducing bulky groups that increase free volume or by enhancing molecular branching. These strategies collectively contribute to lowering the overall polarizability of the material.

The inorganic network formed by vinyl polysiloxane consists of an alternating Si-O silicon-oxygen backbone, which restricts the mobility of polymer chains and thus imparts the composite with excellent overall properties. Furthermore, the large steric hindrance associated with the crosslinked Si-O-Si network limited chain packing density, thereby increasing the free volume of the vinyl polysiloxane and consequently reducing its ε. The high bond energy of Si-O-Si (443.7 kJ/mol) not only effectively lowers the ε within the polymer matrix but also preserves the thermal stability of the resin. The inherently low electronic polarization *α_e_* of vinyl polysiloxane arises from the presence of low-polarity Si-C bonds, which diminish the material’s polarizability. Upon curing, the crosslinked network inhibits both molecular packing and orientational polarization, resulting in reductions in dipole density *N* and distortion polarization *α_d_*. In summary, low-polarity bonds such as Si-O exhibited relatively uniform electron cloud distributions, yielding low electronic polarization *α_e_*. The densely crosslinked structure further restricts polymer chain mobility. With increasing temperature, ongoing hydrosilylation reactions generate Si-C-C-Si linkages with even lower polarity, effectively decreasing electron density, limiting molecular polarization, and reducing chain mobility. This leads to further decreases in *N* and *α_d_*. The orientational polarization *u* was generally minimal in the amorphous state. Collectively, the reductions in *N*, *α_e_*, *α_d_*, and *u* contribute to a low *k*. Such excellent dielectric properties render vinyl polysiloxane precursors highly suitable for radar-transparent material applications.

## 4. Conclusions

This study successfully achieved the goal of developing a high-yield, low-dielectric ceramic precursor with excellent processability for advanced electromagnetic wave-transparent applications; the comprehensive comparison between the PVSO and silica sol is provided in Table 6. The key findings are summarized as follows:

(1) Precursor development: A novel liquid polysiloxane precursor (PVSO) was synthesized via ring-opening polymerization and hydrosilylation. The resulting precursor exhibited excellent rheological properties, including a tunable viscosity of 45–60 mPa·s, which ensured high fluidity and facilitated infiltration-based processing.
(2) Ceramic yield and transformation: Upon pyrolysis in air, PVSO began decomposing at approximately 400 °C and completed conversion at 1000 °C, yielding a ceramic product with a high yield of 81.3%. Elemental analysis confirmed the composition to be nearly pure SiO_2_, with negligible residual carbon, indicating clean and efficient ceramic transformation.
(3) Dielectric performance: The SiO_2_-based ceramic exhibited a dielectric constant of 2.5–2.6 and a loss tangent below 0.01 in the X-band (8.2–12.4 GHz), aligning well with requirements for low-loss electromagnetic applications.
(4) Processing efficiency and application potential: Compared with traditional sol–gel methods, PVSO significantly reduced the number of required infiltration/pyrolysis cycles from over 10 to 6, thereby shortening the fabrication cycle and lowering production costs. These combined advantages position PVSO as a strong candidate for use in radomes, microwave windows, and next-generation high-frequency communication components.

## Figures and Tables

**Table 1:** The ^13^C-NMR chemical shift in PVSO.

Corresponding Functional Group	Chemical Shift
-Si-CH_3_	0.75
-Si-CH_2_-CH_2_-Si-	8.25
-Si-CH_2_-CH_2_-Si-	9.37
-Si-CH(CH_3_)-Si-	1.89
-Si-CH(CH_3_)-Si-	7.82
-Si-CH=CH_2_	134.21
-Si-CH=CH_2_	137.55

**Table 2:** Curing reaction parameters of PVSO at different heating rates.

Heating Rate *Β* (°C/min)	Start Temperature *T_i_* (°C)	Peak Temperature *T_p_* (°C)	Final Temperature *T_f_* (°C)
5	243.75	290.37	319.57
10	260.82	305.61	325.68
15	271.57	312.13	338.72
20	280.01	322.56	342.37

**Table 3:** The exothermal peaks equation of PVSO.

Curing Temperature	Linear Equation	Correlation Coefficient	Static Curing Temperature/°C
*T_i_*	*y* = 234.12 + 2.394*x*	0.98687	234.12
*T_p_*	*y* = 281.89 + 2.061*x*	0.96214	281.89
*T_f_*	*y* = 311.22 + 1.628*x*	0.97836	311.22

**Table 4:** Gas generation during the pyrolysis of PVSO.

*m*/*z*	Temperature (°C)	Gas
2	500–600, 800	H_2_
15, 16	550, 700	CH_4_
18	450	H_2_O
28, 30	400–560	C_2_H_4_
28, 26	400–560	C_2_H_6_
45, 44, 43, 31, 30	400–600	(CH_3_)SiH_2_
59, 58, 45, 44, 43	320, 520	(CH_3_)_2_SiH
73, 59, 42	300–550	(CH_3_)_3_Si

**Table 5:** Elemental composition of PVSO prepared at different temperatures.

Temperature	Si (wt%)	C (wt%)	H (wt%)	O (wt%)
RT	35.433	33.751	5.916	24.9
400 °C	35.251	0.524	1.716	62.509
600 °C	35.414	0.283	1.216	63.087
800 °C	35.414	0.147	0.951	63.488
1000 °C	35.372	0.057	0.432	64.139

**Table 6:** Key differences between PVSO and silica sol routes in ceramic fabrication.

Sample	PVSO	Silica Sol
Ceramic yield (%)	81.3%	≤40%
Molecular designability	High (tunable via Si-H, vinyl, phenyl, etc.)	Low (fixed SiO_2_ composition)
Crosslinking mechanism	Thermal curing (hydrosilylation- or radical-initiated)	Gelation via condensation/polymerization
Final composition	Amorphous or partially crystalline Si-O	Amorphous SiO_2_
Processing complexity	Fewer steps; good shape retention; easy infiltration	Multiple drying/calcination steps; prone to cracking
Microstructure control	Controllable via preceramic polymer architecture	Poor control; aggregation and shrinkage common
Energy consumption	Lower (due to lower pyrolysis temp and shorter cycles)	Higher (due to longer drying and higher sintering temp)
Precursor cost (USD/g)	Moderate (~USD 0.5–1.0/g), with higher yield per unit mass	Low (~USD 0.2–0.4/g), but much lower yield
Cost per g of final ceramic	Lower (~USD 1–1.5/g)	Higher (~USD 2–3/g)

## Data Availability

The original contributions presented in this study are included in the article. Further inquiries can be directed to the corresponding author.

## Nomenclature

The following table summarizes the symbols, abbreviations, and technical terms used throughout the manuscript.

Symbol/Abbreviation	Description
PVSO	Vinyl-Hydrogen Polysiloxane Precursor
D_4_^H^	Tetramethylcyclotetrasiloxane
D_4_^Vi^	Tetravinylcyclotetrasiloxane
TMAH	Tetra-methylammonium hydroxide
FT-IR	Fourier-Transform Infrared Spectroscopy
TGA	Thermogravimetric Analysis
DTG	Derivative Thermogravimetry
SEM	Scanning Electron Microscopy
XRD	X-ray Diffraction
*ρ*	Density (g·cm^−3^)
*η*	Viscosity (mPa·s)
*ε*	Dielectric Constant
tan *δ*	Dielectric Loss Tangent
wt.%	Weight Percent
*T_i_*	Initial Curing Temperature
*T_p_*	Peak Curing Temperature
*T_f_*	Final Curing Temperature
